# Impact of a Reference Center on Leprosy Control under a Decentralized Public Health Care Policy in Brazil

**DOI:** 10.1371/journal.pntd.0005059

**Published:** 2016-10-12

**Authors:** Raquel Rodrigues Barbieri, Anna Maria Sales, Mariana Andrea Hacker, José Augusto da Costa Nery, Nádia Cristina Duppre, Alice de Miranda Machado, Milton Ozório Moraes, Euzenir Nunes Sarno

**Affiliations:** Laboratório de Hanseníase, Instituto Oswaldo Cruz-Fiocruz, Rio de Janeiro, Rio de Janeiro, Brasil; Fondation Raoul Follereau, FRANCE

## Abstract

Reorientation of the public health policies in Brazil over the last 20 years in association with a stable rate of new-case detection prompted the establishment of a decentralized leprosy control strategy. The aim was to move from a vertical model associated with general dermatological services to one in which the diagnosis and treatment of the disease would be integrated into the primary care level of the national health care facilities. Once patients demand for leprosy reference centers began to be affected by the process of integrating leprosy diagnosis into the basic health care services, it was necessary to determine the profile of all our referrals in light of the new decentralization policy.

**Objective:**

We evaluated the profile of patients referred to the Fiocruz Outpatient Clinic, a reference center for the diagnosis and treatment of leprosy in Rio de Janeiro, RJ, and analyzed the origins and outcomes of these referrals.

**Methods:**

This is an observational retrospective study based on information collected from the Leprosy Laboratory database at Fiocruz, Rio de Janeiro, RJ, Brazil. A total of 1,845 suspected leprosy cases examined at the reference center between 2010 and 2014 were included. The originating health service referrals and diagnostic outcomes were analyzed as well as the clinical and epidemiological data of patients diagnosed with leprosy.

**Result:**

Our data show that the profile of the patients treated at the Clinic has changed in recent years. There was an increase in both the proportion of patients with other skin diseases and those who had visited only one health service prior to our Clinic. Among the total 1,845 cases analyzed, the outcomes of 1,380 were linked to other diseases and, in 74% of these cases, a biopsy was not necessary to reach a diagnostic conclusion. A decrease in new leprosy case detection among our patients was also observed. Yet, among the leprosy patients, 40% had some degree of disability at diagnosis.

**Conclusion:**

The results of the present study demonstrated the importance of referral centers in support of basic health services within the decentralization strategy. But, the success of the program depends on the advent of new developmental tools to augment diagnostic accuracy for leprosy. However, it should be emphasized that for new diagnostic methods to be developed, a greater commitment on the part of the health care system regarding research is urgently needed.

## Author Summary

Leprosy, a neglected disease, remains endemic in some developing countries despite the existence of a successful program to treat and cure patients. While has been a drastic decrease in the number of patients, but we still have a stable number of new cases that is still very high in countries like India and Brazil in which more than 30.000 new cases were observed in 2014. Over the past ten years, Brazil has changed the strategies regarding of public health so that leprosy diagnostic, treatment and surveillance functions would predominantly be performed in primary care health units. The decentralization of leprosy diagnosis and treatment was expected to impact early cases detection and contribute to decrease in the number of cases with nerve damage. We analyzed and compared the demand of patient referrals to the Fiocruz Outpatient Clinic, a reference center for the diagnosis and treatment of leprosy in Rio de Janeiro, RJ, Brazil, prior and subsequent to the implementation of the decentralization strategy. Our results indicated that the profile of patients treated at the Fiocruz Clinic changed after the diagnosis and treatment of leprosy was integrated into the primary health services. There was an increase in the proportion of patients with other skin diseases. At the same time, 40% of the patients with leprosy had a higher disability grade at diagnosis, indicating late diagnosis. The initial presentation of leprosy may be a discrete skin or neural lesion, representing a challenge even for trained dermatologists. These results are probably the consequence of difficulties encountered in diagnosing leprosy in the primary health units without specialized health professionals or adequate laboratory tests. Although decentralization strategies have several advantages integrating the diagnosis of leprosy into basic health care units, the support of referral centers in diagnosing complex cases, managing difficult reactional episodes, and treating of side effects is central to the control of the epidemic.

## Introduction

In 1991, the World Health Organization (WHO) adopted the goal of eliminating leprosy as a public health issue worldwide by the year 2000. Elimination was defined as achieving a prevalence rate of lower than 1/10,000 inhabitants [1]. The widespread implementation of the multidrug therapy (MDT) program has been a success, resulting in a substantial reduction in global prevalence. Nonetheless, new case detection rates have not decreased as rapidly as expected in certain countries, especially in Brazil, India, and Indonesia, which have remained endemic. In 2014, 213,899 new leprosy cases were detected around the world while, in the Americas, new cases numbered 33,789, 94% of which were in Brazil [2]. Concurrently in the same year, the detection rate in the State of Rio de Janeiro was 7.36/100,000 inhabitants [3].

Since the advent of the reform within the Brazilian Health System in 1989 and, with it, the initial implementation of the family health strategy, the prevention, diagnosis and treatment of diseases, including leprosy, were moved to the primary care level [4]. Within this system containing an extensive network of health facilities and recognized that the rate of leprosy new case detection has basically not wavered over the last 20 years, it was decided to decentralize leprosy control strategies throughout the country. There was a shift from a vertical model associated with general dermatological services to one in which the diagnosis and treatment of the disease would be integrated into the primary care level facilities. By 2009, the State of Rio de Janeiro had implemented the new strategy in 40% of all its municipal primary health care services [5]. At present, the City of Rio de Janeiro has 147 basic health care that follow the family health model and 59 that are specialized in the traditional sense.

In this context, leprosy reference centers continued to be primarily responsible for diagnosing complex cases, managing difficult reactional episodes, treating the side effects of MDT, evaluating relapse cases and developing research projects [6]. However, once patient demand for leprosy reference centers was affected, in any way, by integrating leprosy diagnosis into basic health care services, it was necessary to determine the pattern of all these referrals in the light of the new decentralization policy.

Previous studies have focused on the consequences of decentralization on the indicators used to evaluate leprosy control, i.e., the proportion of MB patients, new cases under the age of 15, patients diagnosed with physical disabilities as well as on the clinical and epidemiological profiles of leprosy patients [7] [8]. In this study, we decided to investigate the origins and outcomes of suspected leprosy patient referrals to the Fiocruz Outpatient Clinic, a reference center for the diagnosis and treatment of leprosy in Brazil, after the implementation of the decentralization policy.

## Methods

The present study is an observational retrospective study of the information gathered from the Leprosy Outpatient Clinic database at Oswaldo Cruz Foundation (Fiocruz), a reference center for the diagnosis and treatment of leprosy located in the City of Rio de Janeiro, RJ, Brazil.

Patients referred to the Fiocruz Clinic for diagnostic confirmation from January 2010 thru December 2014 were included from the moment we began registering the origin of these referrals in our database. All suspected cases of leprosy who arrived at the Clinic from both public and private health services were included. Likewise, all patients who arrived spontaneously along with the household contacts of new leprosy cases that had some skin or neurological suspicious lesions became participants. Conversely, those who attended at the Clinic for therapeutic management and not for diagnosis (e.g. control of reactions, suspicion of recurrence) and those who abandoned the study prior to receiving a diagnostic conclusion were excluded. The sample under study consisted of 1,845 cases.

The Fiocruz Outpatient Clinic serves individuals from the metropolitan area of the City of Rio de Janeiro as well as other cities in the State. Patients are referred to the Clinic by both public and private health care services, arrive spontaneously, or are household contacts of a leprosy case with suspected skin or neurological lesions.

For diagnostic purposes, patients are routinely undergo dermato-neurological evaluation, skin smears, skin biopsies for histopathological analysis [9] and, if necessary, polymerase chain reaction is performed [10]. Those diagnosed with leprosy are treated and followed up at the Fiocruz Clinic or referred to the original service for treatment. Those diagnosed with other dermatoses or neuropathies are sent to referral services for these specific diseases. Socio-economic, clinical, and epidemiological information, laboratory parameters, and case outcomes are recorded onto a database.

For the present study, the use of these data was approved by the Ethics in Research Committee of the Oswaldo Cruz Foundation number 976.330–10/03/2015.

The variables analyzed in this study were: i) age, sex, place of residence, originating health service (public, private or spontaneous demand), and number of health services the patient consulted before coming to our Clinic (1, 2, 3 or more); ii) clinical diagnosis without a biopsy: leprosy or other disease (OD); iii) histopathological diagnosis: leprosy or other disease; iv) the presence or absence of disability at leprosy diagnosis; and v) patient destination after outcome: continuation at the Fiocruz Clinic, return to the originating service, or referral to another health care service. These clinical and epidemiological aspects of patient referrals during 2010–2014 are presented in the tables; and bivariate analysis were conducted for categorical variables using the chi square test.

Also, in the present study, data during 2005–2014 were analyzed and compared with the 2010–2014. We retrieved data from the official leprosy case data reported by the City and State of Rio de Janeiro obtained at the SINAN (the National Disease Information System) database. We used the following variables: the annual number of new leprosy cases at the Fiocruz Outpatient Clinic and in the City and State of Rio de Janeiro; the annual proportion of newly-diagnosed leprosy cases compared to other diseases at Fiocruz Clinic; the annual percentage of the number of health services visited prior to Fiocruz referrals. Means were calculated to compare the 2005–2009 and 2010–2014 periods.

The statistical analysis was performed using SPSS version 22 software.

## Results

### Descriptive analysis

**Fig 1:**
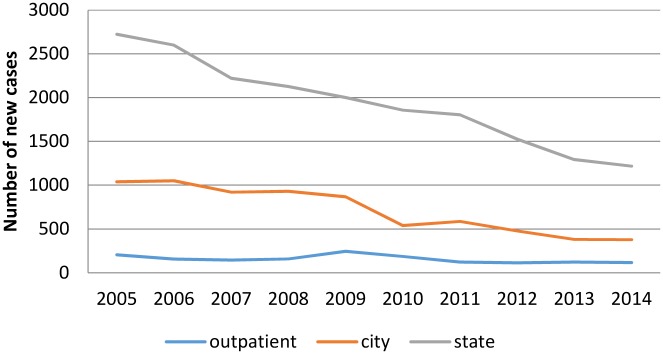
Annual number of new leprosy cases, 2005–2014. Cases diagnosed at the Fiocruz Outpatient Clinic, in City and State of Rio de Janeiro between 2005 and 2014.

The leprosy data regarding both the City and State of Rio de Janeiro reveal an accentuated decline in the number of new leprosy cases between 2005 and 2014, the same being true for the Fiocruz Clinic, as seen in Fig 1. The reduction in the mean of cases between 2010–2014 as compared to 2005–2009 was 51% and 34% in the City and State respectively, while at the Fiocruz Clinic, the decline was lower, 27% (Fig 1).

**Fig 2:**
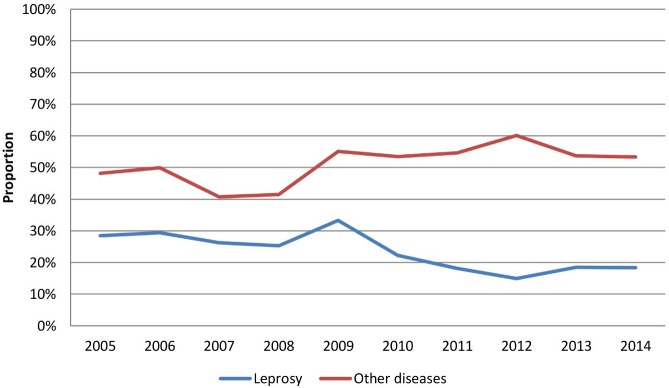
Annual proportion of newly-diagnosed leprosy cases compared to other diseases at the Fiocruz Outpatient Clinic, from 2005 to 2014.

Analysis of total patient demand at the Fiocruz Clinic (new leprosy cases plus those with other skin diseases) showed that the mean proportion of leprosy patients seen at the Fiocruz Clinic between 2005 and 2009 was 28% while the mean proportion of patients with other dermatoses in the same period was 47%. During the 2010–2014, there was an increase of 16% in the proportion of patients with other skin diseases (mean proportion = 55%) compared to the previous period and a proportional decrease of approximately 33% among patients with leprosy (mean proportion = 18%) (p <0.001) (Fig 2).

**Fig 3:**
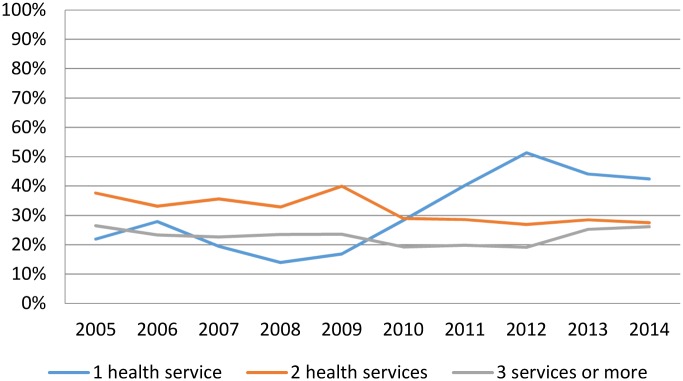
Annual proportion of number of health services prior to patients being referred to the Fiocruz Outpatient Clinic from 2005 to 2014.

Observing that the trend in our Clinic had shifted during 2010–2014 in comparison to 2005–2009, it was decided to evaluate how many health care services had been consulted by these patients before being referred to the Fiocruz Clinic, whose results are showed in Fig 3. The mean proportion of patients who visit only one clinic prior their Fiocruz referral was 20% during 2005–2009 while in the 2010–2014 time period this mean was double (40%) (Fig 3).

Analyzes of the clinical and epidemiological aspects of patients seen after 2010 showed that, of the 1,845 cases evaluated, 1,380 (75%) had other dermatoses or neuropathies and, in 74% of these cases, biopsies were not taken because a leprosy diagnosis could be excluded after a neuro-dermatological evaluation. Only 25% (465) of the cases evaluated at our Clinic for suspected leprosy had a confirmed diagnosis of leprosy. Fifty-one per cent (375) of all the biopsied were diagnosed with leprosy and 49% (359), with other dermatoses (Table 1).

**Table 1:** Evaluated cases of leprosy and other diseases with and without biopsies at the Fiocruz Outpatient Clinic from 2010 to 2014.

Outcome	With biopsy	Without biopsy	Total	*p*
Leprosy	375 (80%)	90 (20%)	465	
Other diseases	359 (26%)	1,021 (74%)	1,380	<0.001
Total	734	1,111	1,845	

Analyzing the association between original referral service and outcome, there was no difference in the positive leprosy outcome between public and private health services referral source. (Table 2). Regarding suspected cases of leprosy who spontaneously arrived at Fiocruz Outpatient Clinic, a significant association with the diagnosis of other diseases was observed. Among these, only 30 (11.3%) were found to have leprosy (Table 2).

**Table 2:** Association between the origin and outcomes of cases referred to Fiocruz Outpatient Clinic from 2010 to 2014.

Origin	Leprosy	Other diseases	Total	*P*
Public health services	300 (26.8%)	817 (73.2%)	1,117	< 0.001
Private health services	123 (28.2%)	313 (71.8%)	436	
Spontaneous demand	30 (11.3%)	236 (88.7%)	266	
**Total**	453*	1,366**	1,819	

*453/465

**1,366/1,380

Table 3 presents the clinical and epidemiological characteristics of cases of confirmed leprosy diagnoses. The majority of patients (70.1%) were between 15 and 59 years of age and 21.6% were over 60. Male patients predominated representing 59% in our cohort. Considering the operational classification, there was a slight difference between the proportion of paucibacillary (PB) versus multibacillary (MB) patients (49% and 51%, respectively) in this study. The disability grade (DG) of two-hundred-and-sixty-two confirmed leprosy patients were registered in the database: 14% had grade 2 of disability and 40% had some degree of disability at diagnosis. There was no statistical significance between the presence of physical disabilities and whether the referrals came from public or private health care facilities. Sixty-nine percent of the diagnosed leprosy patients were treated and followed up at Fiocruz Outpatient Clinic (320/465) (Table 3).

**Table 3:** Epidemiological and clinical aspects of leprosy cases diagnosed at Fiocruz Outpatient Clinic from 2010 to 2014.

Epidemio-clinical variables		n	%
**Age (year)**	>15	38	8.3
	15–29	65	14.2
	30–44	126	27.5
	45–59	130	28.4
	>60	99	21.6
Total		458*	100
**Gender**	Female	170	41
	Male	243	59
Total		413**	100
**Classification**	Paucibacillary	227	49
	Multibacillary	236	51
Total		465	100
**Disability Grade**	0	158	60.3
	1	67	25.6
	2	37	14.1
Total		262***	100
**Destination**	Outpatient Clinic	320	69
	Other services	145	31
Total		465	100

* 7 missing

** 52 missing

*** 203 missing

## Discussion

The results of the present study provide strong evidence that the profile of the patients diagnosed and treated at the Fiocruz Outpatient Clinic have changed in the past 5 years possibly as a result of the public policy shift towards decentralization. The proportion of patients with other skin diseases and those who visited only one health service before our Clinic increased. It is noteworthy that, in 74% of the cases with other diseases, a biopsy was not necessary for diagnosis indicating that general practitioners in the primary care facilities could not distinguish easily diagnosed skin diseases (other than leprosy). After dermatological examination by a specialist at our clinic, the diagnoses of other dermatoses were clearly defined, and were more often eczematous diseases, psoriasis, superficial mycoses or dyschromia (S1 Table).

Nevertheless, a decrease in new leprosy case was not only detected in our Clinic but was also observed in the City and State of Rio de Janeiro. Smith et al. have suggested the possibility that the global decline in case detection in conjunction with the rise in disabilities may be linked to the move from vertical leprosy control activities to integrated approaches [11]. On the other hand, some authors have indicated that the impact of decentralizing policies have pointed to such major gains as reduced prevalence rates, an increase in early detection, and maintenance of the quality of care [12] [13]. Others studies have emphasized the importance of the ability of trained dermatologists to accurately diagnose the disease, suggesting that the greater the success in reducing the disease burden, more important is the role of the specialist with knowledge of the disease and its differential diagnoses [14] [15].

Regarding the epidemiological and clinical aspects of the leprosy cases diagnosed at Fiocruz Outpatient Clinic from 2010 to 2014 there were no important differences concerning age and gender between our results and the proportions recorded in Brazil in 2014. Considering the operational classification, there was a slight difference between the proportion of PB and MB patients (49% and 51%, respectively), while last year, new cases detected in the country was 66% MB [2]. This result may be biased, however, since the differential diagnosis of paucibacillary (PB) leprosy with other dermatoses is often more difficult to achieve than the diagnosis of multibacillary (MB).

Among the leprosy patients, considered in the present study, 40% had some degree of disability at diagnosis. In Brazil, within the leprosy new cases diagnosed in 2014, the percentage of grade 2 disabilities was 6.56% [2]. Both the City and State of Rio de Janeiro have registered rising percentages of this indicator. In fact, the latest five-year averages were 10% and 10.5%, respectively [3]. The Ministry of Health deems a grade 2 disability ≥10% high [16]. It is possible that the fact that this study was carried out in a reference center specialized in the treatment of leprosy had an impact on theirs result due to a selection and measurement biases. However a previous analysis of leprosy patients also treated at the Fiocruz reference center between 2003 and 2007 showed that 12.2% had grade 2 disability and that 32.5% had some degree of disability at diagnosis at that time [17]. These data strongly indicate that the referrals to the Outpatient Clinic of this reference center were, in actuality, delayed, which is particularly surprising in a State with a large number of units and health professionals. On the other hand, this delay may more accurately reflect the difficulties involved in diagnosing leprosy in the primary care health units in the absence of specialized health professionals or laboratory tests to aid in diagnosing the disease, especially since the initial presentation of leprosy may be a slight injury of nerve or a discreet and asymptomatic skin lesion, which would further complicate early diagnosis.

One of the advantages attributed to the integration of leprosy diagnosis and treatment into basic health care units is the increased access of the general population to these services [18] [19] [20]. Theoretically, the integration strategy should contribute to more effective disease control as it would increase the chances of early diagnosis, avoid the occurrence of sequels, break the transmission chain, and increase patient adherence to treatment. But there is a consensus that for integration to be truly successful, health professionals must receive constant theoretical and practical training [19] [20]. A major obstacle is that in many Brazilian states, the permanence of doctors and nurses in primary care health units has proved to be difficult [21] [22]. In the State of Rio de Janeiro, for example, the Public Health Department analyzed the impact of the integration policy for 4 years after its initial implementation into the system. It found that the high professional turnover rate in the primary care health units along with the hardships encountered in obtaining sufficient financial resources to adequately train new professionals are issues that adversely impact the effectiveness of leprosy control measures in the State [18].

In this context, the results of the present study demonstrated the importance of our referral center in support of the basic health care services by accepting cases from all over the State and performing differential diagnosis of skin diseases and neuropathies. Studies performed in other countries have demonstrated that specialized services are necessary and continue to provide significant support within an integrated health care system approach towards the diagnosis and management of leprosy [23] [24].

In addition to medical assistance, reference centers remain committed to their role in developing research that contributes to leprosy control, specifically searching for new tools to more rapidly identify early signs of the disease. For example, many recent reports have shown that PCR-based assays are excellent adjuncts in clinical and histopathological analyses toward the definitive identification of *M*. *leprae* [25] [26]. Other studies aimed at the identification of biomarkers profiles associated with the early onset of type 1 leprosy reactions [27] in addition to antigens that could be used to monitor treatment efficacy in leprosy patients have shown great promise [28]. At specialized leprosy referral centers in Bangladesh and Brazil, Walker et al performed a severity scale for leprosy Type 1 reactions to help diagnose reactional episodes and improve the management of this disabling complication of leprosy [29].

Moreover, the Fiocruz Leprosy Reference Center has developed studies to identify the major risk factors associated with the incidence of leprosy among household contacts in order to support monitoring programs with the use of screening procedures able to spot high-risk individuals thereby widening the opportunities for early diagnosis and treatment. For that purpose, serological test using anti-PGL1 has been performed among leprosy household contacts [30]. In recent years, studies carried out at the Fiocruz Leprosy Laboratory have demonstrated the great value of qPCR in the clinical management of suspected cases of paucibacillary leprosy [10] as well as pure neural leprosy [31]. But, to develop new diagnostic methods, that could be used in a variety of field conditions, to augment diagnostic accuracy, a greater commitment on the part of the health care system regarding research is urgently needed.

## Supporting Information

S1 Table Percentagem of other diseases diagnosed without biopsy at the Fiocruz Outpatient Clinic from 2010 to 2014. (DOCX)

S1 Checklist STROBE checklist. (DOC)

## References

[1] World Health Organization. A Guide to eliminating leprosy as a public health problem. Geneva: World Health Assembly; 1995.

[2] World Health Organization. Weekly Epidemiological Record No. 36, 2015, 90 461–476 http://www.who.int/wer.

[3] State Health Secretary of Rio de Janeiro http://riocomsaude.rj.gov.br/site/Conteudo/Dados.aspx.

[4] Penna MLF, Oliveira MLW, Carmo EH, Penna GO, Temporão JG. The influence of increased access to basic healthcare on the trends in Hansen's disease detection rate in Brazil from 1980 to 2006. *Revista da Sociedade Brasileira de Medicina Tropical*. 2008; 41, 6–10. 19618068 10.1590/s0037-86822008000700003

[5] Valle C, Pimentel M, Libório A, Bittencourt A, Flach D, Saieg F, Mello K. Situação da hanseníase no estado do Rio de Janeiro no período de 2001 a 2009. *Revista Hospital Universitário Pedro Ernesto*. 2011; 10 (1).

[6] Brasil, 2009. Portaria N°. 125/SVS-SAS, de 26 de março de 2009, Brasília-DF Brasil www.credesh.ufu.br/node/1147

[7] Ferreira MLT., Pontes MADA., Silveira MIDS, Araújo LDF, & Kerr LRS. A demanda de um centro de referência nacional para hanseníase no nordeste brasileiro: por que o excesso de pacientes?. *Cad*. *saúde colet*. *(Rio J*.*)*.2008; 16(2).

[8] Mowla MR, Shamim A, and Sanai T. "Leprosy profiles in post ‐ elimination stage: a tertiary care hospital experience." International Journal of Dermatology 2015; 54 12 1407 1413. 10.1111/ijd.12975 26227884

[9] Hacker MDAVB, Sales AM, Albuquerque ECA, Rangel E, Nery JAC, Duppre NC, Sarno E. N. Patients from a reference center for leprosy: Rio de Janeiro and Duque de Caxias, 1986–2008. *Ciência & Saúde Coletiva*. 2012; 17(9), 2533–2541. 22996903 10.1590/s1413-81232012000900033

[10] Barbieri RR, Sales AM, Illarramendi X, Moraes MO, Nery JAC, Moreira SJM et al Diagnostic challenges of single plaque-like lesion paucibacillary leprosy. *Memórias do Instituto Oswaldo Cruz*. 2014; 109(7), 944–947. 10.1590/0074-0276140212 25411000 PMC4296501

[11] Smith WC, van Brakel W, Gillis T, Saunderson P, & Richardus JH. The missing millions: a threat to the elimination of leprosy. *PLoS Negl Trop Dis*. 2015; 9 (4). 10.1371/journal.pntd.0003658 25905706 PMC4408099

[12] Cunha MDD, Cavaliere FAM, Hércules FM, Duraes SMB, Oliveira MLWDR, Matos HJD. The impact of leprosy elimination strategy on an endemic municipality in Rio de Janeiro State, Brazil. *Cadernos de Saúde Pública*. 2007;23(5), 1187–1197. 17486240 10.1590/s0102-311x2007000500020

[13] Kasturiaratchi ND., Settinayake S, Grewal P. Processes and challenges: how the Sri Lankan health system managed the integration of leprosy services. *Leprosy review*. 2002; 73(2), 177–185. 12192974

[14] Oliveira MLW, Penna GO, Telhari S. Role of dermatologists in leprosy elimination and post-elimination era: the Brazilian contribution. *Lepr Rev*. 2007; 78, 17–21. 17518082

[15] Kawuma HJS. Potential role of dermatologists and dermatological services in developing and sustaining the leprosy control referral system in resource constrained settings. *Leprosy review*. 2007; 78(1), 34 17518086

[16] Brasil, 2002. Ministry of Health. Guide to leprosy control. Brasilia: Department of Primary Care, Department of Public Policy; 2002. (Attention Notebooks Basic, 10).

[17] Hacker MA, Sales AM, Illarramendi X, Nery JA, Duppre NC, Bastos FI et al The profile of Patients treated at the national leprosy outpatient referral clinic in Rio de Janeiro, Brazil, 19862007. Rev Panam Salud Publica [Internet]. 6 2012; 31 (6): 485491 22858815 10.1590/s1020-49892012000600006

[18] Pimentel MIF., Andrade M., Valle CLP, Xavier AGM., Bittencourt ALP & Macedo LFSD. Decentralization of the diagnosis and treatment of leprosy in Rio de Janeiro State: advances and problem. *Hansen*. *Int*. 2004; 29(2), 94–100.

[19] Lockwood DNJ and Suneetha S. Leprosy: too complex a disease for a simple elimination paradigm Bull World Health Organ [online] 2005, vol.83, n.3, pp. 230–235. 15798849 PMC2624210

[20] Visschedijk J, Engelhard A, Lever P, Grossi MAF, Feenstra P. Leprosy control strategies and the integration of health services: an international perspective. Cad. Saúde Pública [Internet]. 12 2003; 19 (6): 1567–1581. 10.1590/S0102-311X2003000600002 14999324

[21] Silveira N, Almeida RP. Critical factors for fixing the doctor in the Health Strategy physis Family Public Health Journal, Vol. 22, núm. 4, octubre diciembre, 2012, pp.1293–1311 State University of Rio de Janeiro Rio de Janeiro Brazil.

[22] Medeiros CR, Junqueira AGW, Schwingel G, Carreno I, Jungles LAP, Saldanha OMFL. The turnover of nurses and doctors: a halt in the implementation of the Family Health Strategy Ciênc Public Health. 2010; 15 (suppl 1), 152131. 10.1590/s1413-81232010000700064 20640314

[23] Raffe SF, Thapa M, Khadge S, Tamang K, Hagge D, Lockwood DN. Diagnosis and Treatment of Leprosy Reactions in Integrated Services-The Patients' Perspective in Nepal. *PLoS Negl Trop Dis*. 2013; 7(3), e2089 10.1371/journal.pntd.0002089 23505585 PMC3591330

[24] Wijesinghe PR, Settinayake S. An analysis of the pattern of detection of leprosy patients by institutions in the general health services in Sri Lanka after the integration of leprosy services into general health services. Lepr Rev. 2005; 76: 296–304. 16411509

[25] Williams DL, Scollard DM, Gillis TP. PCR-based diagnosis of leprosy in the United States. *Clinical Microbiology Newsletter*. 2003; 25(8), 57–61. 10.1016/S0196-4399(03)80008-9

[26] Martinez AN, Talhari C, Moraes MO, Talhari S. PCR-Based Techniques for Leprosy Diagnosis: From the Laboratory to the Clinic. PLoS Negl Trop Dis. 2014; 8(4): e2655 10.1371/journal.pntd.0002655 24722358 PMC3983108

[27] Khadge S, Banu S, Bobosha K, van der Ploeg-van JJ, Goulart IM, Thapa P et al Longitudinal immune profiles in type 1 leprosy reactions in Bangladesh, Brazil, Ethiopia and Nepal. *BMC infectious diseases*. 2015; 15(1), 1 10.1186/s12879-015-1128-0 26510990 PMC4625471

[28] Spencer JS, Duthie MS, Geluk A, Balagon F, Kim HJ, Wheat WH et al Identification of serological biomarkers of infection, disease progression and treatment efficacy for leprosy. Mem. Inst. Oswaldo Cruz.Vol. 2012; 107(Suppl. I): 79–89. 10.1590/S0074-02762012000900014 23283458

[29] Walker SL, Nicholls PG, Butlin CR, Nery JAC, Roy HK, et al Development and Validation of a Severity Scale for Leprosy Type 1 Reactions. PLoS Negl Trop Dis. 2008; 2(12): e351 10.1371/journal.pntd.0000351 19104651 PMC2596969

[30] Düppre NC, Camacho LA, Sales AM, Illarramendi X, Nery JA, Sampaio EP, Sarno EN, Bührer-Sékula S: Impact of PGL-I seropositivity on the protective effect of BCG vaccination among leprosy contacts: a cohort study. PLoS Negl Trop Dis. 2012, 6 (6): e1711 10.1371/journal.pntd.0001711 22724040 PMC3378622

[31] Jardim MR, Antunes SL, Simons BRIAN, Wildenbeest JG, Nery JAC, Illarramendi X & Sarno EN. Role of PGL-I antibody detection in the diagnosis of pure neural leprosy. *Leprosy review*, 2005; 76(3), 232–240. 16248210

