# Supplementary material for: Impact of a Reference Center on Leprosy Control under a Decentralized Public Health Care Policy in Brazil
Source: PLoS Negl Trop Dis. 2016 Oct 12;10(10):e0005059. doi: 10.1371/journal.pntd.0005059 (PMC5061346; doi:10.1371/journal.pntd.0005059)
Supplement: S1 Table — (DOCX) [file pntd.0005059.s001.docx]

Table S1: Percentagem of other diseases diagnosed without biopsy at the Fiocruz Outpatient Clinic from 2010 to 2014

| **Diseases** | **n** | **%** |
| --- | --- | --- |
| Eczematous disease/Psoriasis | 134 | 13.1 |
| Neuropathies | 119 | 11.6 |
| Superficial and subcutaneous mycoses | 87 | 8.5 |
| Dyschromia | 44 | 4.3 |
| Pharmacodermias | 25 | 2.4 |
| Rheumatologic diseases | 24 | 2.3 |
| Benign and malignant tumors | 22 | 2.1 |
| Granuloma annulare | 19 | 1.8 |
| Piodermitis | 15 | 1.4 |
| Erythema Multiforme / Erythema nodosum | 15 | 1.4 |
| Infectious granulomatous diseases | 11 | 1.0 |
| Others skin diseases | 323 | 31.6 |
| **Total** | 838 | 100 |

*183 outcomes are missing at the Fiocruz database
